# Diagnosis Prediction of Lumbar Spinal Canal Stenosis by Plain Radiography Using Discriminative Radiographic Features

**DOI:** 10.3390/diagnostics16213544

**Published:** 2026-10-28

**Authors:** Tzu-Ping Yu, Che-Wei Lin, Hao-Chun Chuang, Nurul Maulidiyah, Chien-Hsiung Lo, Yuan-Fu Liu, Chao-Jui Chang, Yu-Meng Hsiao, Cheng-Li Lin

**Affiliations:** 1 Department of Orthopedic Surgery, National Cheng Kung University Hospital, College of Medicine, National Cheng Kung University, Tainan 704, Taiwan; g860920@gmail.com (T.-P.Y.); link.m50801@gmail.com (H.-C.C.); david811912@gmail.com (C.-H.L.); andwantfly@gmail.com (Y.-F.L.); vx9527@hotmail.com (C.-J.C.); 2 Department of Biomedical Engineering, College of Engineering, National Cheng Kung University, Tainan 701, Taiwan; lincw@mail.ncku.edu.tw (C.-W.L.); nurul.maulidiyah@bm.itera.ac.id (N.M.); 3 Institute of Gerontology, College of Medicine, National Cheng Kung University, Tainan 701, Taiwan; 4 Medical Device Innovation Center, National Cheng Kung University, Tainan 701, Taiwan; 5 Department of Orthopedics, Dou-Liou Branch of National Cheng Kung University Hospital, Yunlin 640, Taiwan; 6 Department of Orthopedics, Tainan Municipal An-Nan Hospital, China Medical University, Tainan 709, Taiwan; jasonmunmun1@gmail.com; 7 Musculoskeletal Research Center, Innovation Headquarter, National Cheng Kung University, Tainan 701, Taiwan

**Keywords:** lumbar spinal canal stenosis, plain radiography, magnetic resonance imaging, prediction, machine learning

## Abstract

**Purpose:** To investigate whether widely available lumbar spine radiography can predict lumbar spinal canal stenosis (LSCS). **Methods:** This retrospective, single-center cross-sectional study included patients who underwent both lumbar spine radiography and magnetic resonance imaging. Correlations between radiographic and MRI parameters and their associations with LSCS were analyzed with adjustment for age and sex. The predictive performance of machine learning algorithms was evaluated with nested cross-validation. **Results:** Among 201 subjects, 104 had L4/5 LSCS and 97 did not; the LSCS group was 18 years older. After adjustment for age and sex, static spondylolisthesis (aOR 5.62, *p* < 0.001), average disc height (aOR 1.23, *p* = 0.021), and dynamic lordotic angle (aOR 0.96, *p* = 0.011) remained significant predictors, and adding radiographic parameters to age and sex increased the AUC from 0.85 to 0.91 (*p* = 0.007). Static spondylolisthesis remained significant in an age- and sex-matched subset (aOR 4.81). In nested cross-validation, machine learning models combining radiographic parameters with age and sex achieved an accuracy of 80.7% (sensitivity 82.9%, specificity 78.4%, AUC 0.87), compared with 76.2% for radiographic parameters alone. **Conclusions:** Lumbar spine radiography provides information on LSCS that is additional to age. Radiographic signs reflecting the stabilization phase of spinal degeneration, particularly static spondylolisthesis and decreased dynamic lordosis, were the most robust predictors. External validation is required before clinical use.

## 1. Introduction

Lumbar spinal canal stenosis (LSCS), caused by reduced spinal canal area, has become the most common spinal degenerative disease in elderly patients [1]. The patients usually have pain, numbness, weakness in the lower back or extremities, and may even suffer from neurogenic claudication or functional disability. Generally, the lesions comprise disc herniation, osteophyte formation, facet joint hypertrophy or ligamentum flavum hypertrophy (LFH). Amidst all, LFH contributes a crucial proportion of LSCS due to its considerable occupying area in the spinal canal. The etiology of LFH is thought to be multifactorial, including aging, mechanical stress, increased inflammation, and the elevated oxidative stress, etc. Refs. [2,3].

In clinical practice, the diagnosis of LSCS relies on clinical assessments and imaging confirmation. The image can provide accurate morphological information for clinical judgement [4]. Plain radiography is usually the first image taken for patients with symptoms of LSCS. For detailed evaluation, magnetic resonance imaging (MRI) is considered the primary choice due to its soft tissue contrast. However, MRI is not easily available in every facility or medical system. Besides, the cost and waiting time have raised great concern for timely intervention. On the contrary, plain radiography is an inexpensive, informative, and highly accessible imaging modality. Previous studies identified the relationship between the parameters of computed tomography (CT) and LFH [5]. However, few studies have investigated the correlation between the radiographic parameters and LSCS [6]. In addition, the recent advances in artificial intelligence techniques offer promising applications in medical imaging [7,8]. Therefore, the current study aimed to identify and validate the potential of the plain radiographic signs for the prediction of LSCS diagnosis by combining statistical analysis and machine learning-based approaches. Such a model is intended to help prioritize patients for MRI rather than to replace it.

## 2. Materials and Methods

### 2.1. Participants

**Figure 1:**
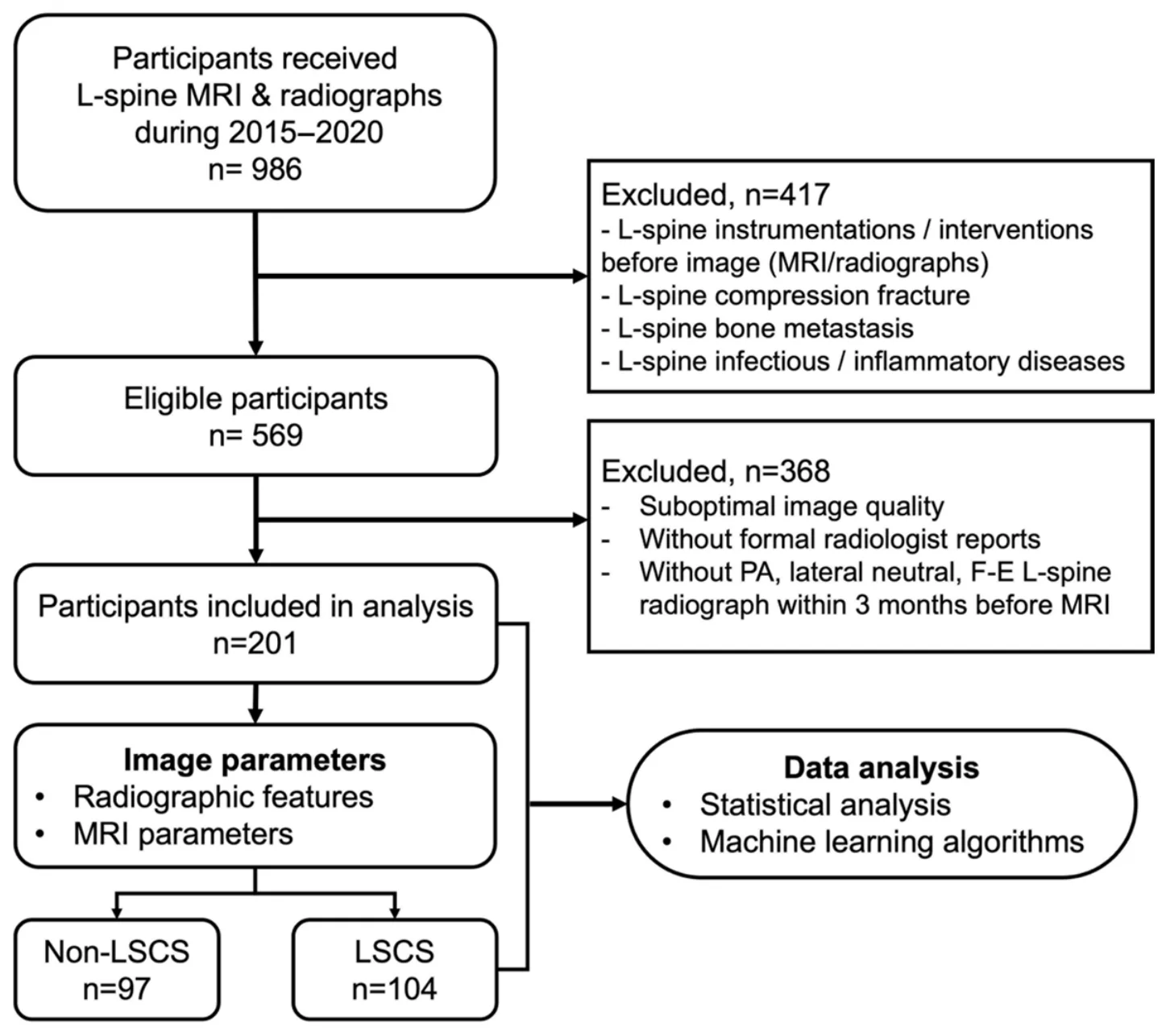
Flow chart diagram of study design. The labeled image parameters and the clinical diagnosis underwent both conventional statistical analysis and machine learning-based calculation. MRI: Magnetic Resonance Imaging, L-spine: lumbar spine, LSCS: lumbar spinal canal stenosis, PA: posteroanterior, F-E: flexion–extension.

In this retrospective, single-center study, we enrolled patients who underwent both lumbar spine radiography and MRI at a tertiary center, National Cheng Kung University Hospital (NCKUH) in Taiwan between 2015 and 2020. L4/5 was chosen as the index level because it is the segment most frequently affected by degenerative central canal stenosis, and restricting the analysis to one segment allowed each segmental radiographic parameter to be paired with the MRI measurements at the same level. Patients with clinical symptoms related to lumbar symptomatology at a spine level of L4/5 and who underwent both lumbar spine radiography and MRI within 3 months after admission were included. Patients with a history of previous lumbar spinal surgery or interventions, compression fracture, malignancy, metastasis, congenital anomalies, discitis, osteomyelitis and ankylosing spondylitis were excluded. Based on the recorded medical charts, patients were divided into LSCS and non-LSCS groups (Figure 1).

### 2.2. Diagnosis of Lumbar Spinal Canal Stenosis

Experienced spine surgeons evaluated LSCS by both clinical and radiological diagnostic criteria. The clinical criteria included patients with presence of low back pain, lower limbs radicular pain or weakness, or intermittent claudication [4,9]. These symptoms would mostly be relieved by flexing forward, exacerbated while walking and persisted for months despite non-operative management. The radiological criteria vary among radiologists with no consistent objective parameters (e.g., ligamentum flavum thickness above 4.0 mm, ligamentum flavum area above 105.9 mm2, dural sac area below 100 mm^2^) and often need to correlate with patients’ clinical presentations [5,10]. In this study, no single quantitative MRI threshold was applied; LSCS at L4/5 was diagnosed by the treating spine surgeons when typical symptoms were accompanied by central canal narrowing at L4/5 on MRI, and the thresholds above are cited for reference only. The surgeons had access to all routine imaging, including the plain radiographs, when assigning the diagnosis; that is, the reference standard was not blinded to the radiographs.

### 2.3. Evaluation of Lumbar Spinal Canal Stenosis on MRI

The parameters representing the spinal canal stenosis at the L4/5 segment were measured on axial images of T2-weighted MRI at the facet joint level (Supplementary Figure S1A). The cross-sectional area of ligamentum flavum (LFA) was also measured by drawing a line along the side of the ligament facing the spinal canal and along the laminar side of the ligament margin (Supplementary Figure S1B). Subsequently, dural sac area (DSA), which was proposed to be highly correlated with LSCS, was outlined (Supplementary Figure S1C) [11,12]. Then, LFA divided by DSA was also calculated. Measurements were carried out twice, and the mean values were recorded.

### 2.4. Radiographic Parameters of Lumbar Spinal Instability and Motion

Sagittal angular change and the translational difference on the flexion–extension (dynamic) views were recorded for assessment of spinal instability. The sagittal angular change could also be used to evaluate the motion of the spine [13]. The intradiscal angle was measured between the inferior endplate of the superior vertebra and the superior endplate of the inferior vertebra (Supplementary Figure S2A). The segmental angle was measured between the superior endplate of the superior vertebra and the inferior endplate of the inferior vertebra (Supplementary Figure S2B). The lumbar lordotic angle was measured between the tangent lines that were drawn along the superior endplate of L1 and S1 (Supplementary Figure S2C). The sacral slope was the angle of the sacral plateau to the horizontal (Supplementary Figure S2D). The absolute difference of these angles between the dynamic films was recorded as dynamic angles (lordosis was given positive; conversely, kyphosis was given negative). Sagittal translation was obtained as the difference between the displacements of L4/5 vertebrae on the dynamic views (anterolisthesis was given positive; conversely, retrolisthesis was given negative).

### 2.5. Radiographic Parameters of Lumbar Spinal Degeneration

Disc height, vacuum disc phenomenon, and disc calcification were identified to evaluate intervertebral disc degeneration. Anterior, posterior, and average disc height (average of anterior and posterior disc height) were determined as the distance between the endplates of adjacent vertebrae on the lateral radiograph. The vacuum phenomenon was evaluated by the presence of gaseous radiolucency in the disc space (Supplementary Figure S3A) [13]. The presence of calcium density within the intervertebral space was defined as disc calcification. Patients with distinct sclerotic bands presented at the bony endplates were recorded as endplate sclerosis. Osteophyte formation was defined as bony protrusions or outgrowth from the vertebral rim (Supplementary Figure S3B). Facet arthropathy was noted if marginal osteophytes, subchondral sclerosis, or irregular joint surfaces were seen (Supplementary Figure S3C) [14]. Bony defects or fractures within the pars interarticularis were recorded as spondylolysis (Supplementary Figure S3C). Abnormal spinal curvature with a Cobb angle > 10° on the anteroposterior view was recorded as degenerative scoliosis [15]. The degenerative spondylolisthesis was recorded using the Meyerding classification (Supplementary Figure S3D), and further divided into dynamic and static types. Dynamic spondylolisthesis was defined as the sagittal translation difference greater than 3 mm between the dynamic films [16].

### 2.6. Statistical Analysis

For the univariate between-group comparisons, power analysis was performed using G*Power software (version 3.1.9.7; Heinrich-Heine-Universität Düsseldorf, Düsseldorf, Germany) with a two-tailed independent *t* test (α = 0.05, power = 0.80). Assuming a medium effect size (Cohen’s d = 0.5), a minimum of 64 patients per group (128 in total) was required. The enrolled sample (104 LSCS and 97 non-LSCS patients) exceeded this requirement. For the multivariable models, sample size was assessed using the criteria of Riley et al. [17]. Assuming an outcome prevalence of 51.7% (104/201), seven predictor parameters in model 3, and a conservative Cox–Snell R^2^ of 0.35 (approximated from the C statistic of 0.85 for age and sex alone), at least 149 patients were required (expected shrinkage ≥ 0.90 and optimism in Nagelkerke R^2^ ≤ 0.05), which the 201 enrolled patients exceeded. The criterion for precise estimation of the overall outcome proportion (margin of error ≤ 0.05) would have required 384 patients; with 201 patients, the achieved margin of error was approximately ±0.07. With 97 patients in the smaller outcome group, model 3 (the five radiographic parameters of model 2 plus age and sex) had approximately 14 events per predictor parameter, whereas model 1 (13 parameters) had approximately 7 and was therefore regarded as exploratory [17]. Because the radiographic predictors were selected stepwise from 13 candidates, events per candidate parameter were approximately 6.5 (97/15, including age and sex); this risk of overfitting was addressed by nested cross-validation (Section 2.7) and is noted as a limitation.

All radiographic parameters underwent univariate analysis with MRI parameters. Quantitative variables were presented as correlation coefficients (r) and analyzed by Pearson correlation. Binary radiographic variables were presented as mean ± standard deviation (SD) and analyzed by independent Student’s *t*-test. Two-tailed *p*-values < 0.05 were considered significant. Univariate logistic regressions were performed to estimate the odds ratio (OR) and 95% confidence interval (CI) of radiographic parameters for LSCS. In model 1, statistically significant parameters were included to examine the relative contribution of each parameter to the outcomes. In model 2, variables with *p* < 0.05 were analyzed by the forward stepwise method. After each entry, variables in the model were tested based on the significance of the conditional statistic criterion. The ultimate model was chosen in accordance with the highest area under the curve (AUC) calculated by receiver operating characteristic (ROC) analysis. Inter-observer reliability was assessed for 25 randomly selected patients measured independently by two observers, using a two-way random-effects, absolute-agreement, single-measure intraclass correlation coefficient (ICC) with 95% CI. The data above were analyzed using SPSS version 22.0 (IBM Corp., Armonk, NY, USA).

To address the age difference between the groups, model 2 was additionally adjusted for age and sex (model 3), and its discrimination was compared with that of a model containing age and sex alone (model 0) using the likelihood-ratio test and DeLong’s test for correlated ROC curves. As a sensitivity analysis, patients with and without LSCS were matched 1:1 on sex and age (caliper ± 3 years) by greedy nearest-neighbor matching without replacement, and model 2 was refitted in the matched subset; calipers of 5 years and matching on age alone were also examined. These additional analyses were performed in Python 3.12 with statsmodels 0.15.0.

### 2.7. Machine Learning

**Figure 2:**
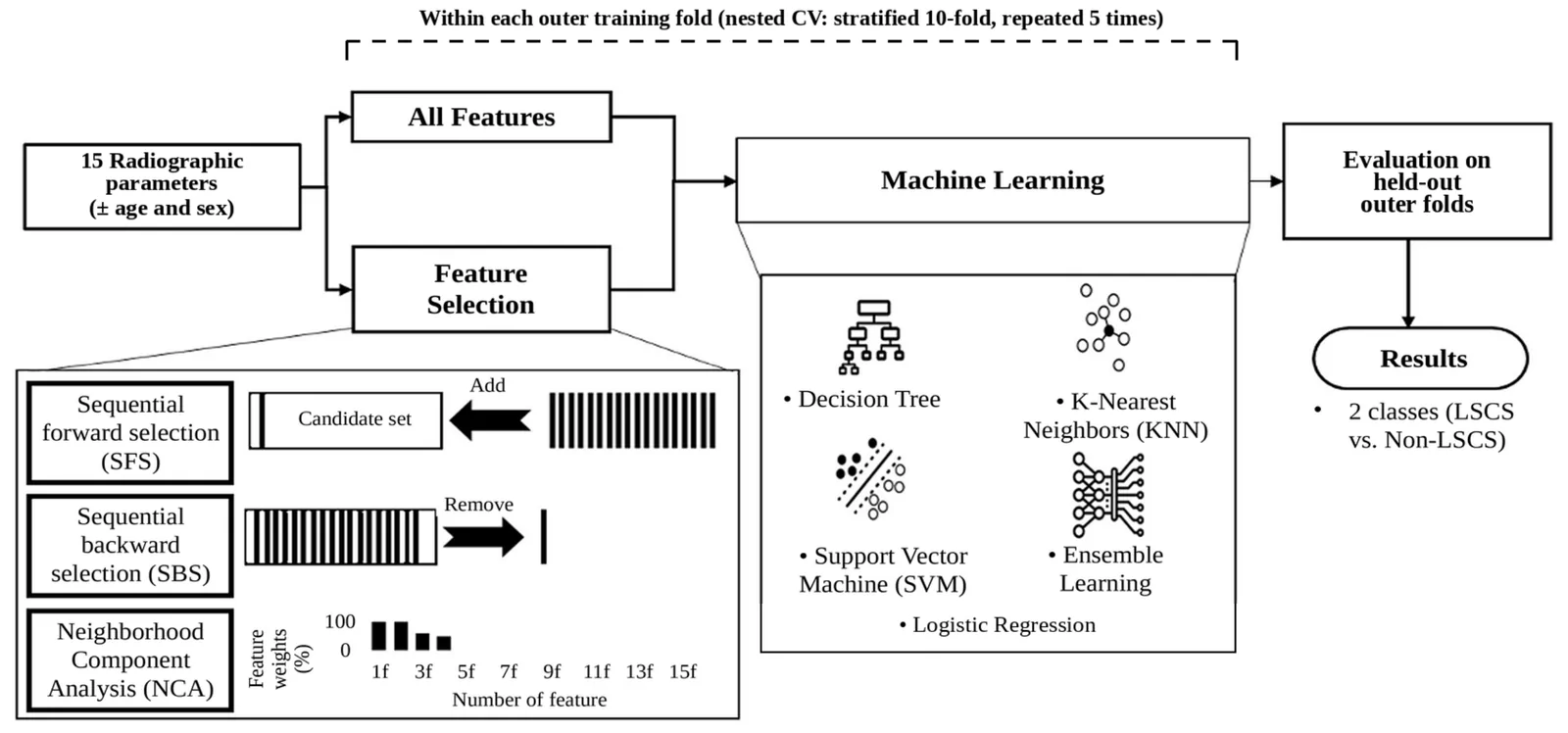
Flow chart of LSCS classification based on a machine learning approach. In total, 15 radiographic parameters, with or without age and sex, were entered as candidate inputs of the prediction model. Sequentially, feature selection (SFS, SBS, NCA; only NCA was used within the nested cross-validation), machine learning algorithms (decision tree, KNN, SVM, ensemble learning, logistic regression), and validation were performed; feature selection and model tuning were nested within the training folds of a repeated stratified 10-fold cross-validation (Section 2.7), which served as the primary validation, to classify the diagnosis (LSCS or non-LSCS). Model performance was evaluated only on the held-out outer folds, which were not used for feature selection or model tuning; the original, non-nested comparison using leave-one-out cross-validation (LOOCV) is described in Section 2.7 for reference only. The dashed bracket indicates the steps repeated within each outer training fold. SFS: sequential forward selection, SBS: sequential backward selection, NCA: neighborhood component analysis, SVM: support vector machine, KNN: k-nearest neighbors, CV: cross-validation, LSCS: lumbar spinal canal stenosis.

Machine learning of LSCS classification algorithm with radiographic parameters was used to classify the patients’ diagnosis (LSCS or non-LSCS) (Figure 2). Sequential feature selection was used to decrease the initial dimensions of measured features and obtain the best feature subset to achieve greater classification quality. Sequential forward selection (SFS) [18,19], sequential backward selection (SBS) [18], and neighborhood component analysis (NCA) [20] were the three variations of this methodology. SFS involved gradual addition of the features to an empty candidate set and testing each step until addition no longer improved the model’s misclassification rate [19]. SBS methodology began with the entire candidate set and then iteratively eliminated the least contributing features one at a time [18]. NCA divided the multivariate data into different classes based on the distance measurement over the data and selected the optimum feature subset by maximizing the average LOO (leave-one-out) classification performance over the training data [20]. In this study, NCA was used only for feature ranking (its internal leave-one-out objective is part of the NCA algorithm and is distinct from model validation), and both the ranking and the number of retained features were determined within the training data of each cross-validation fold, as described below.

Selected features were inserted into the algorithms for machine learning by the strategy of decision tree, support vector machine (SVM), k-nearest neighbor (KNN), and ensemble learning. A decision tree was made of nodes representing the predictor variables that connected to two or more sub-trees and leaves to determine which node would profit the most [18]. SVM employed the kernel function to convert the input data space into a higher-dimensional data space to distinguish classifiers and was explicitly specified by a separating hyperplane. It generated an ideal separation hyperplane between the two classes in the converted space [21]. In KNN, an object was classified by majority vote of its neighbors, and consequently assigned to the most common class among its KNNs. By comparing the observations in the test set to the training dataset, it could categorize them depending on the number of neighbors [22]. The ensemble learning method built a model with basic classifiers and merged their judgments to make the final prediction. These ensemble learners could avoid the overfitting of input data as well as the presence of noise in the data [23]. Logistic regression was additionally included as a linear reference classifier, and a random forest of bagged decision trees was used for ensemble learning.

To avoid information leakage from feature selection, model performance was estimated with nested cross-validation. The outer loop was stratified 10-fold cross-validation repeated five times with different random partitions. Within each outer training fold only, missing values were imputed with the median, features were standardized, the NCA regularization parameter (11 values between 0 and 20/n) was selected by inner stratified 5-fold cross-validation, features were ranked by their NCA weights, and the number of top-ranked features was selected for each classifier by inner 5-fold cross-validated accuracy. The classifier was then trained on the outer training fold and evaluated on the held-out outer fold, which was not used in any step of feature selection or model tuning. All 15 radiographic parameters were entered as candidates; dynamic spondylolisthesis was not entered separately because it is a dichotomized form of sagittal translation (>3 mm), which was included as a continuous variable. Three feature sets were evaluated: radiographic parameters alone, radiographic parameters with age and sex, and age and sex alone. Accuracy, sensitivity, specificity, and AUC were calculated from the pooled out-of-fold predictions of each repetition and are reported as mean ± standard deviation across repetitions, and the frequency with which each parameter was selected across the 50 outer folds was recorded. For comparison, the original non-nested procedure, in which NCA was applied to the full dataset before leave-one-out cross-validation (LOOCV) [24], was also reproduced; this LOOCV estimate is reported only to illustrate the optimistic bias of non-nested feature selection and was not used as the primary validation. The analyses were performed in Python 3.12 with scikit-learn 1.8.0, and NCA followed the feature-weighting formulation of Yang et al. [20].

In clinical terms, the procedure comprised three steps. First, 15 radiographic parameters that can be read on routine anteroposterior, lateral, and flexion–extension radiographs were entered as candidates, with or without age and sex. Second, using only the training part of each cross-validation split, the parameters were ranked by how well they separated patients with and without LSCS, and the number of top-ranked parameters was chosen. Third, the resulting model was tested on the held-out patients, who had not been used in any step of model building. Because this is performed within the same cohort, it represents internal validation only.

## 3. Results

After exclusion (Figure 1), 201 participants, 111 men and 90 women, were included. Among them, 104 patients were diagnosed with LSCS at L4/5 and 97 patients were not, with mean ages of 65.58 ± 10.11 years and 47.92 ± 14.77 years (*p* < 0.001), respectively. The MRI parameters representing LSCS (LFA, DSA, LFA/DSA) differed significantly between the LSCS and non-LSCS groups (*p* < 0.001) (Table 1). Inter-observer reliability was excellent (ICC 0.97, 95% CI: 0.92–0.98; *n* = 25).

### 3.1. The Relationship Between Plain Radiographic and MRI Parameters Related to LSCS

Table 2 shows the relationship between radiographic and MRI parameters. Increased average disc height positively correlated with LFA and LFA/DSA. Average vertebral body height was negatively correlated with DSA but positively associated with LFA/DSA. Dynamic angular change showed a negative correlation with LFA and LFA/DSA and a positive correlation with DSA. All MRI parameters showed significant differences between those with and without vacuum disc, osteophyte formation, and static spondylolisthesis.

### 3.2. The Relationship Between Plain Radiographic Parameters and LSCS Diagnosis

The univariable and multivariable logistic regression for all the radiographic parameters and LSCS diagnosis was shown in Table 3. Increased average disc height, decreased dynamic angles and spondylosis signs (except dynamic spondylolisthesis) increased the risk of LSCS. After enrolled the significant parameters in model 1 and 2, average disc height, facet arthropathy, and static spondylolisthesis were still significant. AUC in model 2 reached 0.86 (95% CI: 0.81–0.91), which was close to the AUC of model 1 (AUC: 0.87; 95% CI: 0.83–0.92). Because the LSCS group was considerably older, model 2 was further adjusted for age and sex (model 3; Table 4). Static spondylolisthesis (aOR 5.62, 95% CI 2.25–13.99, *p* < 0.001), average disc height (aOR 1.23, 95% CI 1.03–1.47, *p* = 0.021), and dynamic lordotic angle (aOR 0.96, 95% CI 0.92–0.99, *p* = 0.011) remained significant, whereas degenerative scoliosis (*p* = 0.261) and facet arthropathy (*p* = 0.264) did not. Age and sex alone yielded an AUC of 0.85 (95% CI 0.80–0.90), comparable to that of model 2 (*p* = 0.778). Adding the radiographic parameters to age and sex significantly improved model fit (likelihood-ratio χ2 = 37.3, df = 5, *p* < 0.001) and discrimination (AUC 0.91, 95% CI 0.87–0.95; ΔAUC 0.056, 95% CI 0.016–0.096, *p* = 0.007). In the age- and sex-matched subset (49 pairs; mean age 59.3 vs. 58.5 years, *p* = 0.662), static spondylolisthesis (aOR 4.81, 95% CI 1.72–13.43, *p* = 0.003) and average disc height (aOR 1.28, 95% CI 1.03–1.59, *p* = 0.027) remained significant, dynamic lordotic angle was of borderline significance (*p* = 0.050), and model 2 achieved an AUC of 0.77 (95% CI 0.67–0.87). Static spondylolisthesis remained significant under all alternative matching criteria (calipers of 3 or 5 years, with or without sex matching; aOR 3.42–5.21).

### 3.3. Prediction of LSCS Diagnosis by Radiographic Parameters

**Figure 3:**
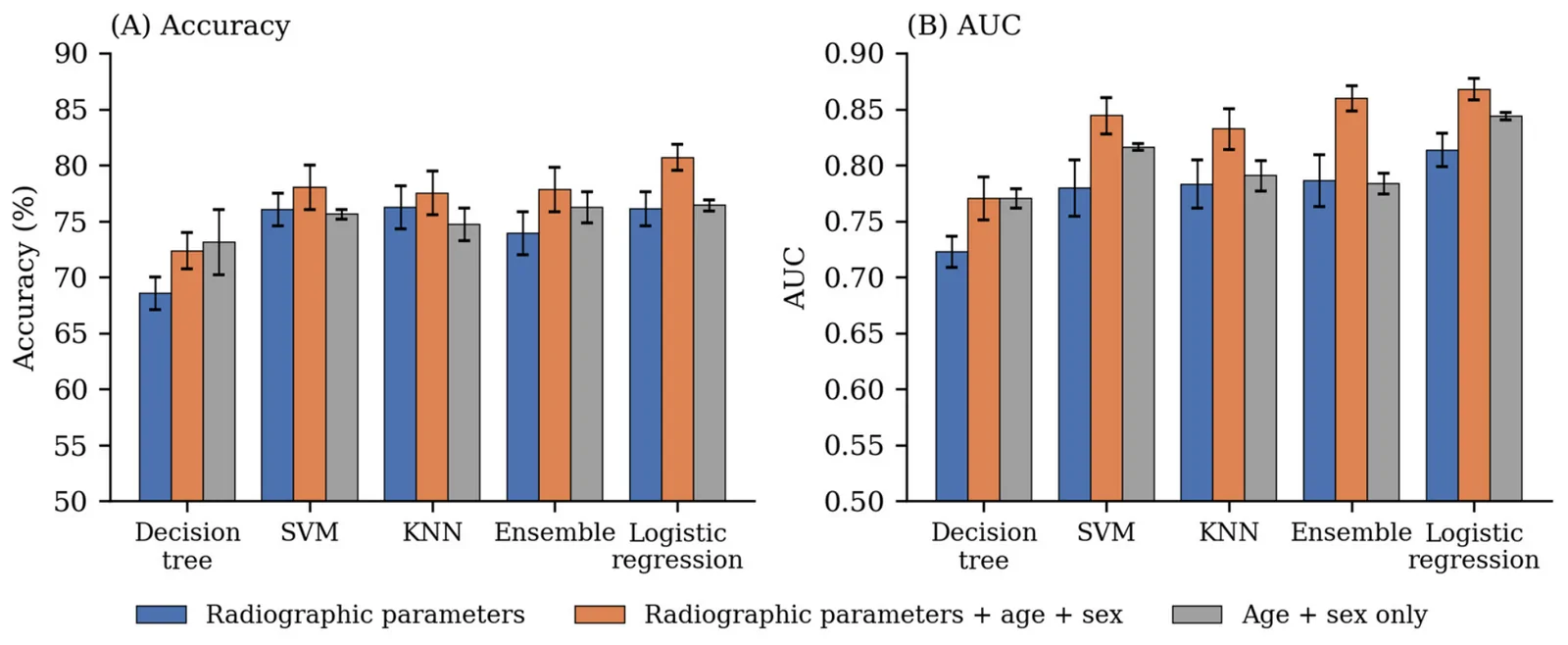
Nested cross-validated performance of machine learning algorithms. Bars show the mean (**A**) accuracy and (**B**) area under the curve (AUC) of five repetitions of stratified 10-fold cross-validation for radiographic parameters alone, radiographic parameters with age and sex, and age and sex alone; error bars indicate the standard deviation across repetitions. Feature selection by NCA and the number of selected features were determined within each training fold. SVM: support vector machine, KNN: k-nearest neighbors, NCA: neighborhood component analysis, AUC: area under the curve.

The selection frequency of each radiographic parameter across the outer folds is shown in Table 5, and the nested cross-validated performance of the machine learning algorithms is shown in Table 6 and Figure 3. Using radiographic parameters alone, the best accuracy was 76.2% ± 1.9% (KNN; sensitivity 75.6%, specificity 76.9%), and the highest AUC was 0.81 ± 0.01 (logistic regression). Combining radiographic parameters with age and sex yielded the best overall performance (logistic regression: accuracy 80.7% ± 1.2%, sensitivity 82.9%, specificity 78.4%, AUC 0.87 ± 0.01), compared with an accuracy of 76.4% and an AUC of 0.84 for age and sex alone. For SVM, KNN, ensemble learning, and logistic regression, adding the radiographic parameters to age and sex increased the mean AUC by 0.02–0.08, and the increase was observed in every repetition. Static spondylolisthesis and dynamic lordotic angle were selected in 100% and 88–100% of outer folds, respectively, followed by degenerative scoliosis (48–90%) and facet arthropathy (34–90%), whereas all other parameters were selected in ≤52% of folds (Table 5). The median number of selected radiographic parameters was three to six. For reference, reproducing the original non-nested procedure on the same data yielded a maximum accuracy of 80.1%, indicating that the previously reported accuracy of 82.6% was optimistically biased by feature selection on the full dataset.

## 4. Discussion

The current study showed that plain radiographic signs could help with a fast prediction of LSCS. Multivariate analysis showed that LSCS diagnosis was associated with average disc height, dynamic lordotic angle, degenerative scoliosis, static spondylolisthesis, and facet arthropathy. The AUC of the five combined factors was 0.86. Machine learning analysis suggested static spondylolisthesis, dynamic lordotic angle, degenerative scoliosis, and facet arthropathy were the top four factors in LSCS prediction. After adjustment for age and sex, static spondylolisthesis, average disc height, and decreased dynamic lordosis remained independent predictors, and the radiographic parameters improved discrimination beyond age and sex (AUC from 0.85 to 0.91). Because age alone discriminated the groups nearly as well as the radiographic model, radiographic signs should be interpreted together with age rather than as a substitute for it. Typical symptomatology of LSCS, with the signs mentioned above, provided a fast screening of LSCS.

LSCS has been considered as an advanced manifestation of degenerative spondylosis related to aging [1,2]. Diagnosis of LSCS generally requires clinical evaluation combined with imaging evidence, particularly MRI [1,4]. However, it is very difficult to arrange MRI for all patients. Thus, other modalities including CT, CT myelography, or plain radiography are also used for interpretation [6,25]. Amidst all, plain radiography is the most available image for patients encountering lumbar symptomatology. It was reported that the discovery of specific radiographic features suggesting LSCS could markedly support the clinical judgement [6].

Lumbar spinal degenerative diseases appeared to evolve in three phases: early dysfunction, instability, and stabilization [26]. The early dysfunction phase predominantly started with intervertebral disc degeneration [27]. Increased strain on the intervertebral disc caused the development of clefts or fissures and led to disc herniation, the intradiscal vacuum phenomenon, or calcification [26,27]. As intervertebral disc degeneration proceeded, the spine gradually lost support and progressed to the instability phase. Structures other than the intervertebral disc bore the greater mechanical load to maintain the alignment [13]. The extra load would greatly transmit to the facet joints and cause destruction, characterized by fibrocartilaginous metaplasia, marginal osteophytes, or subchondral calcification [28]. Besides, the long-term multidirectional instability creates rotational or translational subluxation, resulting in degenerative scoliosis, spondylolisthesis, and osteophytes [29]. Eventually, instability triggered the compensatory stabilizing response and entered the stabilization phase. Hypertrophic facet joint osteoarthritis and significant LF hypertrophy might occur in this stage [2,3]. Besides, the spondylolisthesis transferred from dynamic to static type [26]. Overall, the spinal canal would be narrowed by virtue of surrounding hypertrophic tissues or displaced structures. Thus, decreased lumbar motion and spinal canal stenosis might develop in patients deemed to have advanced degenerative diseases.

The current study identified the parameters of plain radiography representing each degenerative phase. Features representing the last stabilization phase (static spondylolisthesis, decreased lumbar motion) instead of earlier phases were correlated with both MRI parameters and clinical diagnosis of LSCS. However, the parameters correlated with MRI parameters and diagnosis were slightly mismatched. The possible reason for this could be stated as that the diagnosis contained clinical evaluations and lateral recess or foraminal stenosis, which could not be detected by the MRI parameters chosen in this study.

Compared to previous studies which focused on the relationship between CT or MRI parameters and the diagnosis [5,12], we extended the concepts to a further dimension since plain radiography is more approachable. Previously, disc degeneration and severe facet joint osteoarthritis were correlated with the LF thickness on MRI [5], and these parameters were similar to our findings, which indicated the late stage of spinal degeneration. Compared to LF thickness as the characterization of LSCS, the cross-sectional area might be more representative [10,11]. Decreased DSA was thought to be a core feature of LSCS [11,12], and others have proposed that increased LFA was associated with LSCS [30]. This brought forth the idea of the ratio of LFA to DSA, which was more indicative of evidence of spinal canal stenosis and could standardize individual differences.

Because patients with LSCS were on average 18 years older than those without, part of the apparent predictive value of degenerative radiographic signs could reflect age rather than stenosis itself. Indeed, age and sex alone achieved an AUC of 0.85, which was not significantly different from that of the unadjusted radiographic model (0.86; DeLong *p* = 0.78). After adjustment for age and sex, facet arthropathy and degenerative scoliosis were no longer independently associated with LSCS, suggesting that their crude associations were largely age-related. In contrast, static spondylolisthesis, greater average disc height, and reduced dynamic lordotic motion remained independent predictors after adjustment; static spondylolisthesis also remained significant in an age- and sex-matched subset, and the radiographic parameters added significant discrimination beyond age and sex (AUC 0.85 to 0.91). These signs therefore carry information about L4/5 stenosis that is not explained by patient age, and in practice, they should be interpreted together with age rather than in place of it.

In practice, the model is intended as a triage aid rather than a replacement for MRI. For a patient with typical symptoms, the presence of static spondylolisthesis and reduced dynamic lordotic motion on routine radiographs, interpreted together with age, indicates a higher probability of L4/5 stenosis and may justify earlier MRI, whereas their absence should not be used to exclude LSCS. Compared with the simplest clinical information, age and sex, the radiographic signs added significant discrimination. The machine learning classifiers did not clearly outperform conventional logistic regression; their main contribution was to confirm, by an independent data-driven method, that a small set of radiographic signs carries most of the predictive information.

In Table 3, increased average disc height was significantly associated with the diagnosis of LSCS. This finding is counterintuitive, because disc height generally decreases with degeneration, and may partly reflect measurement error. In this study, average disc height was measured from the plain radiograph by measuring the average distance between two vertebral bodies. Although this method is straightforward, it may overestimate the true disc height. Choi et al. [31] reported that the distance measured by this method was much longer than that from CT scan. Another method provided by Frobin et al. [32] might reveal the real disc height. Nevertheless, average disc height remained independently associated with LSCS after adjustment for age and sex (Table 4). It was therefore retained in the model, but its odds ratio should be interpreted with caution given this measurement limitation, and it should not be used as a stand-alone predictor. Compared to disc height, the sagittal translation and tilt between two vertebral bodies, which cause spinal canal narrowing, can be detected more precisely by plain radiography. Besides, average disc height was selected in only 10–44% of the outer cross-validation folds (Table 5), much less frequently than the first four factors.

In addition to the statistical analysis, machine learning-based frameworks were performed to sort out the generalizable predictive patterns. This strategy had been used in other spine diseases [8,33,34]. The features that machine learning chose were surprisingly consistent with the statistical results. When feature selection was nested within cross-validation, the estimated accuracy for radiographic parameters alone decreased from the previously reported 82.6% to 76.2%, which underlines the importance of strictly separating feature selection from performance evaluation in small datasets. This implied that the crucial features were not only correlated but also regarded as predictive markers for the clinical diagnosis. Thus, it was suggested that by thoroughly recording the radiographic features, representative MRI parameters, clinical diagnoses from specialists, and applying diverse analytic methods, this study would provide more comprehensive perspectives of LSCS.

However, this study had several limitations. First, this was a retrospective, single-center study; the patient population, referral pattern, radiographic protocol, and diagnosing surgeons all came from one tertiary hospital, so the findings may not be generalizable to other settings. Second, our sample size was limited. Although the sample met the criteria of Riley et al. for the seven parameters of model 3 [17], it did not meet them when the 15 candidate parameters screened by stepwise selection were considered (approximately 6.5 events per candidate parameter), so some overfitting of the multivariable models cannot be excluded, and the overall outcome proportion was estimated less precisely than recommended (384 patients would have been required for a margin of error of ≤ 0.05; the achieved margin was approximately ±0.07). Furthermore, the degenerative radiographic parameters are correlated with one another, and the number of events per variable was limited for Model 1, whose estimates should be regarded as exploratory. In addition, the image diagnostic criteria remain debated, and the clinical relationship between radiographic parameters and LSCS has not yet been verified. Moreover, the diagnosing surgeons were not blinded to the plain radiographs, so the radiographic predictors may have partly contributed to the reference standard (incorporation bias). The non-LSCS group was also on average 18 years younger than the LSCS group, which may have introduced spectrum bias; although the main findings persisted after adjustment for age and sex and in an age- and sex-matched subset, the matched analysis included only 49 pairs. Further, apart from age and sex, we did not include the patient’s clinical characteristics in the predictive model owing to the design of the radiographical study. This might be less thorough for the diagnosis, which is worthy of future study. Only the L4/5 level was analyzed; because other levels, particularly L5/S1, differ in alignment, mobility, and pattern of stenosis, the findings cannot be extrapolated to them without level-specific validation. Most importantly, model performance was assessed only by internal (nested cross-) validation in this cohort, and external validation in an independent, preferably multicenter, cohort with a comparable age distribution is required before clinical use. Lastly, the application of this study varies across medical systems with the divergent accessibility of MRI. Under the framework of National Health Insurance in our country, it is challenging to arrange timely MRI for all suspicions. Nonetheless, this study may still provide novel and credible concepts on the diversity of LSCS diagnosis.

## 5. Conclusions

In conclusion, distinct features on plain radiography, especially in the stabilization phase of lumbar spinal degeneration, were identified with a strong relationship with MRI parameters and clinical diagnosis of LSCS. These features provided diagnostic information beyond age and sex, with static spondylolisthesis being the most robust predictor, and machine learning models combining radiographic features with age and sex achieved an accuracy of 80.7% in nested cross-validation. These results provide physicians with timely evaluation and supporting information for the diagnosis of LSCS. While it is recommended that all patients should undergo MRI for a definitive diagnosis of LSCS, the constraints of limited accessibility and resources prioritize patients with a higher probability of LSCS based on plain radiography. Because the models were only internally validated in a single-center cohort, external validation is required before clinical application.

## Figures and Tables

**Table 1:** Demographic data and MRI parameters.

	All (*n* = 201)	LSCS (*n* = 104)	Non-LSCS (*n* = 97)	*p*-Value
Demographic				
Age (yr)	57.05 ± 15.35	65.58 ± 10.11	47.92 ± 14.77	**<0.001**
Sex (M:F)	111:90	49:55	62:35	**0.023**
MRI parameters				
LFA (mm^2^)	129.96 ± 26.69	142.44 ± 25.84	116.58 ± 20.44	**<0.001**
DSA (mm^2^)	142.67 ± 60.08	120.93 ± 53.27	165.97 ± 58.44	**<0.001**
LFA/DSA	1.12 ± 0.65	1.44 ± 0.73	0.79 ± 0.29	**<0.001**

Significant results are shown in bold. The values above are given as the mean and the standard deviation. LSCS: lumbar spinal canal stenosis, yr: years, M: male, F: female, LFA: ligamentum flavum area, DSA: dural sac area.

**Table 2:** Relationship between the radiographic and MRI parameters.

Radiographic Parameters	Mean ± SD or N	LFA		DSA		LFA/DSA	
		r or Mean ± SD	*p*-Value	r or Mean ± SD	*p*-Value	r or Mean ± SD	*p*-Value
**Quantitative parameters**							
Avg. disc height (mm)	9.26 ± 2.50	0.15	**0.040**	−0.12	0.091	0.18	**0.009**
Avg. vertebral body height (mm)	30.17 ± 3.03	0.07	0.304	−0.15	**0.032**	0.16	**0.021**
Sagittal translation (mm)	2.56 ± 2.91	0.02	0.815	−0.05	0.526	−0.01	0.916
Dynamic intradiscal angle (°)	6.30 ± 5.19	−0.23	**0.001**	0.15	**0.029**	−0.26	**<0.001**
Dynamic segmental angle (°)	8.25 ± 7.50	−0.14	**0.043**	0.15	**0.032**	−0.18	**0.009**
Dynamic lordotic angle (°)	32.39 ± 13.79	−0.30	**<0.001**	0.25	**<0.001**	−0.32	**<0.001**
Dynamic sacral slope (°)	16.70 ± 18.71	−0.14	**0.048**	0.11	0.122	−0.17	**0.013**
**Spondylosis signs**							
Vacuum disc			**<0.001**		**<0.001**		**<0.001**
Positive	36	143.89 ± 28.37		112.67 ± 44.14		1.55 ± 0.85	
Negative	165	126.92 ± 25.40		149.21 ± 61.02		1.03 ± 0.56	
Endplate sclerosis			**0.004**		0.084		**0.004**
Positive	25	144.24 ± 30.30		123.25 ± 57.31		1.47 ± 0.76	
Negative	176	127.93 ± 25.59		145.43 ± 60.11		1.07 ± 0.62	
Disc calcification			**0.010**		0.073		0.055
Positive	12	149.23 ± 29.42		112.48 ± 33.39		1.47 ± 0.65	
Negative	189	128.74 ± 26.12		144.59 ± 60.94		1.10 ± 0.65	
Osteophyte formation			**<0.001**		**0.003**		**<0.001**
Positive	114	135.98 ± 27.37		131.72 ± 60.88		1.29 ± 0.72	
Negative	87	122.07 ± 23.70		157.01 ± 56.19		0.91 ± 0.47	
Facet arthropathy			0.335		0.063		**0.020**
Positive	135	131.24 ± 27.52		137.16 ± 60.18		1.20 ± 0.70	
Negative	66	127.36 ± 24.90		153.95 ± 58.72		0.97 ± 0.51	
Spondylolysis			**0.017**		0.890		0.095
Positive	33	140.07 ± 24.95		144.00 ± 68.70		1.30 ± 0.82	
Negative	168	127.98 ± 26.64		142.41 ± 58.47		1.09 ± 0.61	
Degenerative scoliosis			0.311		0.273		0.164
Positive	46	133.47 ± 30.22		134.12 ± 55.69		1.24 ± 0.75	
Negative	155	128.92 ± 25.56		145.21 ± 61.27		1.08 ± 0.62	
Dynamic spondylolisthesis			0.721		0.818		0.618
Positive	91	130.70 ± 24.47		141.59 ± 56.89		1.10 ± 0.57	
Negative	110	129.35 ± 28.49		143.56 ± 82.84		1.15 ± 0.71	
Static spondylolisthesis			**0.005**		**<0.001**		**<0.001**
Positive	66	137.50 ± 26.42		121.38 ± 61.21		1.47 ± 0.79	
Negative	135	126.28 ± 26.13		153.08 ± 56.90		0.96 ± 0.49	

Significant results are shown in bold. For quantitative parameters, r is the Pearson correlation coefficient; for spondylosis signs, MRI parameters were compared between patients with and without the sign by independent *t*-test, and the *p*-value is shown once in the row of each sign. SD: standard deviation, LFA: ligamentum flavum area, DSA: dural sac area, Avg.: average.

**Table 3:** The effect of the radiographic parameters for diagnosis of LSCS in univariate and multivariate analysis.

Radiographic Parameters	Univariate			Model 1			Model 2		
	OR	95% CI	*p*-Value	aOR	95% CI	*p*-Value	aOR	95% CI	*p*-Value
**Quantitative parameters**									
Avg. disc height (mm)	1.18	1.05–1.33	**0.006**	1.21	1.04–1.42	**0.017**	1.22	1.05–1.43	**0.011**
Avg. vertebral body height (mm)	1.05	0.96–1.15	0.321						
Sagittal translation (mm)	0.98	0.89–1.07	0.609						
Dynamic intradiscal angle (°)	0.89	0.84–0.95	**<0.001**	0.97	0.88–1.06	0.459			
Dynamic segmental angle (°)	0.92	0.88–0.96	**<0.001**	0.99	0.94–1.05	0.751			
Dynamic lordotic angle (°)	0.93	0.90–0.95	**<0.001**	0.97	0.93–1.00	0.075	0.94	0.91–0.97	**<0.001**
Dynamic sacral slope (°)	0.96	0.94–0.98	**<0.001**	0.98	0.96–1.01	0.123			
**Spondylosis signs**									
Vacuum disc	4.10	1.76–9.53	**0.001**	1.82	0.57–5.87	0.313			
Endplate sclerosis	8.41	2.43–29.11	**0.001**	1.58	0.32–7.74	0.570			
Disc calcification	11.36	1.44–89.71	**0.021**	1.79	0.16–20.46	0.642			
Osteophyte formation	2.70	1.52–4.80	**0.001**	0.91	0.40–2.05	0.810			
Facet arthropathy	5.22	2.72–10.05	**<0.001**	2.40	1.05–5.46	**0.037**	2.55	1.16–5.57	**0.019**
Spondylolysis	2.47	1.11–5.51	**0.027**	0.98	0.28–3.44	0.974			
Degenerative scoliosis	4.61	2.14–9.94	**<0.001**	2.70	0.98–7.43	0.054	2.91	1.14–7.40	**0.025**
Dynamic spondylolisthesis	0.85	0.49–1.48	0.555						
Static spondylolisthesis	8.78	4.20–18.33	**<0.001**	6.59	2.75–15.82	**<0.001**	6.40	2.78–14.74	**<0.001**
**AUC**				0.87	0.83–0.92		0.86	0.81–0.91	

Significant results are shown in bold. AUC is not applicable to the univariate analyses. LSCS: lumbar spinal canal stenosis, Avg: average, aOR: adjusted odds ratio, CI: confidence interval, AUC: Area under the curve.

**Table 4:** Age- and sex-adjusted logistic regression model and age- and sex-matched sensitivity analysis for the diagnosis of LSCS.

Parameters	Model 3 (*n* = 201)			Age- and Sex-Matched Subset (49 Pairs)		
	aOR	95% CI	*p*-Value	aOR	95% CI	*p*-Value
Age (yr)	1.09	1.05–1.13	**<0.001**	–	–	–
Male sex	0.37	0.16–0.87	**0.023**	–	–	–
Avg. disc height (mm)	1.23	1.03–1.47	**0.021**	1.28	1.03–1.59	**0.027**
Dynamic lordotic angle (°)	0.96	0.92–0.99	**0.011**	0.96	0.92–1.00	0.050
Static spondylolisthesis	5.62	2.25–13.99	**<0.001**	4.81	1.72–13.43	**0.003**
Degenerative scoliosis	1.80	0.65–5.05	0.261	1.79	0.52–6.12	0.354
Facet arthropathy	1.67	0.68–4.10	0.264	1.72	0.61–4.79	0.303
AUC (95% CI)	0.91	0.87–0.95		0.77	0.67–0.87	

Model 3 includes the five radiographic parameters of model 2 together with age and sex. Age and sex alone (model 0) yielded an AUC of 0.85 (95% CI 0.80–0.90); adding the radiographic parameters increased the AUC by 0.056 (95% CI 0.016–0.096; DeLong *p* = 0.007; likelihood-ratio χ^2^ = 37.3, df = 5, *p* < 0.001). In the matched subset (1:1 matching on sex and age within ±3 years; mean age 59.3 vs. 58.5 years, *p* = 0.662), model 2 was refitted without age and sex. Significant results are shown in bold. LSCS: lumbar spinal canal stenosis, Avg.: average, aOR: adjusted odds ratio, CI: confidence interval, AUC: area under the curve.

**Table 5:** Selection frequency of radiographic parameters across the outer folds of nested cross-validation (radiographic parameters only).

Radiographic Parameters	Selection Frequency (%)				
	Decision Tree	SVM	KNN	Ensemble	Logistic Regression
Static spondylolisthesis	100	100	100	100	100
Dynamic lordotic angle (°)	88	98	100	98	98
Degenerative scoliosis	62	48	72	90	50
Facet arthropathy	66	46	78	90	34
Spondylolysis	22	22	24	52	14
Dynamic sacral slope (°)	18	12	22	52	14
Avg. disc height (mm)	18	16	20	44	10
Sagittal translation (mm)	22	12	2	44	8
Avg. vertebral body height (mm)	18	8	4	34	6
Dynamic segmental angle (°)	6	10	0	30	6
Osteophyte formation	16	2	0	26	0
Vacuum disc	12	4	2	24	2
Endplate sclerosis	8	4	2	24	4
Disc calcification	6	0	0	28	2
Dynamic intradiscal angle (°)	10	4	0	18	2

Values are the percentage of the 50 outer training folds (stratified 10-fold cross-validation repeated five times) in which the parameter was among the top-ranked NCA features retained for each classifier. Feature ranking and the number of retained features were determined within each outer training fold only. NCA: neighborhood component analysis, SVM: support vector machine, KNN: k-nearest neighbors, Avg.: average.

**Table 6:** Nested cross-validated performance of machine learning algorithms for the classification of diagnosis (LSCS or non-LSCS).

Input Features	Algorithm	Accuracy (%)	Sensitivity (%)	Specificity (%)	AUC	No. of Features (Median)
Radiographic parameters	Decision tree	68.6 ± 1.5	63.5 ± 2.5	74.0 ± 2.2	0.72 ± 0.01	4
	SVM	76.0 ± 1.5	75.2 ± 2.8	76.9 ± 1.2	0.78 ± 0.03	3
	KNN	**76.2 ± 1.9**	75.6 ± 2.2	76.9 ± 2.0	0.78 ± 0.02	4
	Ensemble learning	73.9 ± 1.9	75.2 ± 3.3	72.6 ± 2.1	0.79 ± 0.02	6
	Logistic regression	76.1 ± 1.5	76.2 ± 1.9	76.1 ± 1.5	0.81 ± 0.01	3
Radiographic parameters + age + sex	Decision tree	72.3 ± 1.6	69.8 ± 1.8	75.1 ± 4.3	0.77 ± 0.02	5
	SVM	78.0 ± 2.0	78.5 ± 2.5	77.5 ± 2.6	0.84 ± 0.02	9.5
	KNN	77.5 ± 1.9	74.6 ± 3.7	80.6 ± 2.6	0.83 ± 0.02	6
	Ensemble learning	77.8 ± 2.0	79.4 ± 2.3	76.1 ± 2.4	0.86 ± 0.01	12
	Logistic regression	**80.7 ± 1.2**	82.9 ± 1.7	78.4 ± 2.0	0.87 ± 0.01	5
Age + sex only	Decision tree	73.1 ± 2.9	72.3 ± 3.4	74.0 ± 2.6	0.77 ± 0.01	2
	SVM	75.6 ± 0.4	74.6 ± 1.0	76.7 ± 1.1	0.82 ± 0.00	2
	KNN	74.7 ± 1.5	73.1 ± 2.6	76.5 ± 0.4	0.79 ± 0.01	2
	Ensemble learning	76.2 ± 1.4	78.3 ± 2.0	74.0 ± 2.6	0.78 ± 0.01	2
	Logistic regression	**76.4 ± 0.5**	77.7 ± 0.7	75.1 ± 0.4	0.84 ± 0.00	2

Values are mean ± standard deviation across five repetitions of stratified 10-fold cross-validation. Feature selection by NCA and the number of selected features were determined within each outer training fold. The highest accuracy for each input feature set is shown in bold. For reference, the original non-nested procedure (NCA applied to the full dataset followed by LOOCV) yielded a maximum accuracy of 80.1% on the same data. LSCS: lumbar spinal canal stenosis, SVM: support vector machine, KNN: k-nearest neighbors, AUC: area under the curve, NCA: neighborhood component analysis, LOOCV: leave-one-out cross-validation.

## Data Availability

The de-identified patient-level data and the analysis code supporting the findings of this study are available from the corresponding author on reasonable request. The data are not publicly available because they consist of patient-level clinical imaging measurements.

## Supplementary Materials

The following supporting information can be downloaded at: https://www.mdpi.com/article/10.3390/diagnostics16213544/s1, Figure S1: Measurements of lumbar spinal canal stenosis on MRI; Figure S2: Measurements of lumbar spinal angles on plain radiography; Figure S3: Assessment of degenerative lumbar spine on plain radiography.

## Abbreviations

The following abbreviations are used in this manuscript:

LSCS	Lumbar spinal canal stenosis
LFH	Ligamentum flavum hypertrophy
LFA	Ligamentum flavum area
MRI	Magnetic resonance imaging
CT	Computed tomography
NCKUH	National Cheng Kung University Hospital
DSA	Dural sac area
CI	Confidence interval
AUC	Area under the curve
ROC	Receiver operating characteristic
ICC	Intraclass correlation coefficient
SFS	Sequential forward selection
SBS	Sequential backward selection
NCA	Neighborhood component analysis
SVM	Support vector machine
KNN	K nearest neighbor
LOOCV	Leave-one-out cross-validation
