# Supplementary material for: Diagnosis Prediction of Lumbar Spinal Canal Stenosis by Plain Radiography Using Discriminative Radiographic Features
Source: Diagnostics (Basel). 2026 Oct 28;16(21):3544. doi: 10.3390/diagnostics16213544 (PMC13762167; doi:10.3390/diagnostics16213544)
Supplement: Supplementary file 1 [file diagnostics-16-03544-s001.zip › diagnostics-4560388-supplementary.pdf]

Supplementary figures:

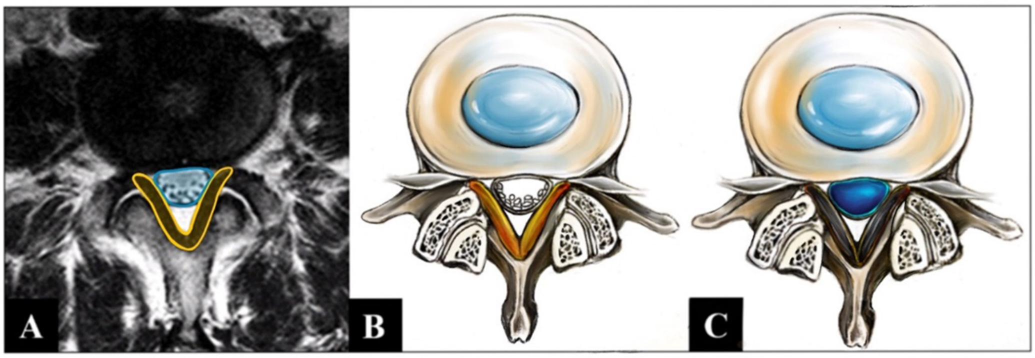

Figure S1. Measurements of lumbar spinal canal stenosis on MRI. Panel A: T2-weighted axial view of lumbar spine at facet joints level, Panel B: Colored area (orange) is LFA, Panel C: Colored area (dark blue) is DSA. ||LFA: ligamentum flavum area, DSA: dural sac area.

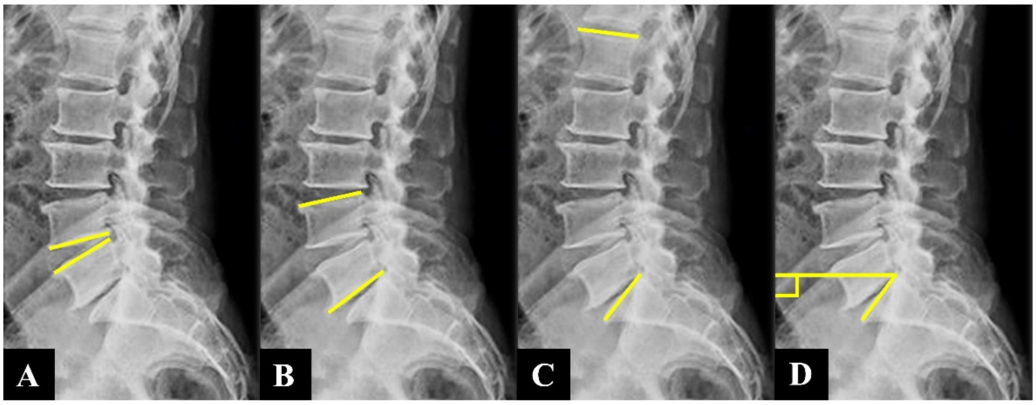

Figure S2. Measurements of lumbar spinal angles on plain radiography. The angles were calculated between the two yellow lines marked on the lateral radiographs. Panel A: Intradiscal angle, Panel B: Segmental angle, Panel C: Lordotic angle, Panel D: Sacral slope.

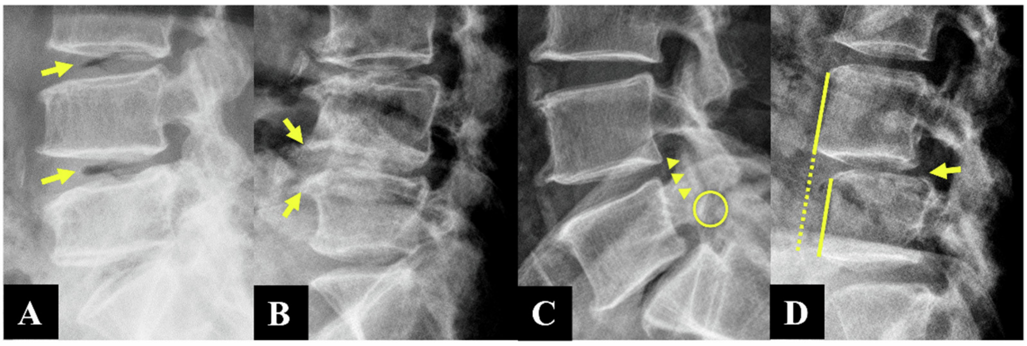

Figure S3. Assessment of degenerative lumbar spine on plain radiography. Panel A: Intradiscal vacuum phenomenon, Panel B: Tractional osteophytes on vertebral rim, Panel C: Facet arthropathy-subchondral sclerosis (arrow), Spondylolysis (circle), Panel D: Spondylolisthesis (arrow).
